# Questionable value of [^99m^Tc]-sestamibi scintigraphy in patients with pHPT and negative ultrasound

**DOI:** 10.1007/s00423-022-02648-9

**Published:** 2022-08-09

**Authors:** Christina Lenschow, Andreas Wennmann, Anne Hendricks, Christoph-Thomas Germer, Martin Fassnacht, Andreas Buck, Rudolf A. Werner, Lars Plassmeier, Nicolas Schlegel

**Affiliations:** 1 Department of General, Visceral, Transplant, Vascular and Pediatric Surgery, University Hospital Würzburg, Oberduerrbacherstrasse 6, 97080 Würzburg, Germany; 2 Department of Internal Medicine I, Division of Endocrinology and Diabetes, University Hospital Würzburg, Oberduerrbacherstrasse 6, 97080 Würzburg, Germany; 3 Department of Nuclear Medicine, University Hospital Würzburg, Oberduerrbacherstrasse 6, 97080 Würzburg, Germany

**Keywords:** Primary hyperparathyroidism, Parathyroid adenoma, [^99m^Tc]-Sestamibi scan, [^11^C]-Methionine, [^11^C]-Choline PET/CT, Focused surgical approach

## Abstract

**Purpose:**

A successful focused surgical approach in primary hyperparathyroidism (pHPT) relies on accurate preoperative localization of the parathyroid adenoma (PA). Most often, ultrasound is followed by [^99m^Tc]-sestamibi scintigraphy, but the value of this approach is disputed. Here, we evaluated the diagnostic approach in patients with surgically treated pHPT in our center with the aim to further refine preoperative diagnostic procedures.

**Methods:**

A single-center retrospective analysis of patients with pHPT from 01/2005 to 08/2021 was carried out followed by evaluation of the preoperative imaging modalities to localize PA. The localization of the PA had to be confirmed intraoperatively by the fresh frozen section and significant dropping of the intraoperative parathyroid hormone (PTH) levels.

**Results:**

From 658 patients diagnosed with pHPT, 30 patients were excluded from the analysis because of surgery for recurrent or persistent disease. Median age of patients was 58.0 (13–93) years and 71% were female. Neck ultrasound was carried out in 91.7% and localized a PA in 76.6%. In 23.4% (135/576) of the patients, preoperative neck ultrasound did not detect a PA. In this group, [^99m^Tc]-sestamibi correctly identified PA in only 25.4% of patients. In contrast, in the same cohort, the use of [^11^C]-methionine or [^11^C]-choline PET resulted in the correct identification of PA in 79.4% of patients (OR 13.23; 95% CI 5.24–33.56).

**Conclusion:**

[^11^C]-Methionine or [^11^C]-choline PET/CT are superior second-line imaging methods to select patients for a focused surgical approach when previous ultrasound failed to identify PA.

## Introduction

Primary hyperparathyroidism is the third most common endocrine disorder which is caused by one or several hyperfunctioning parathyroid glands [1]. The only curative treatment is surgical removal of the hyperfunctioning glands which is successful in > 97% of all cases [1, 2]. Nowadays, pHPT is most often diagnosed following randomly detected hypercalcemia in routine checks or when patients show up with typical clinical symptoms referred to as kidney stones, reduced bone density or fractures, or gastrointestinal disorders [1]. Meanwhile, there is also evidence that psychological and neurocognitive alterations are common symptoms [3, 4]. Whereas the indication for surgery is unanimous seen in the latter group, there is some debate, if all patients with proven pHPT benefit from surgery [1, 5–7]. In general, the diagnosis of pHPT is based on an elevated serum calcium adjusted for albumin in the presence of an elevated or inappropriately normal intact PTH after exclusion of a familial hypocalciuric hypercalcemia [1, 5].

It is well established that the most successful diagnostic tool (> 95%) to detect parathyroid adenoma (PA) is the surgeon during cervical exploration [8]. Nonetheless, preoperative parathyroid imaging has become a standard procedure to locate abnormal parathyroid tissue. A common approach is that following neck ultrasound [^99m^Tc]-sestamibi scintigraphy is performed to confirm the identification of the PA or to exclude an aberrant localization before bilateral surgical exploration is intended [9]. In cases where localization diagnostics do not lead to the successful detection of PA, a bilateral surgical exploration can be carried out. Alternatively, it may be discussed with the patient to perform extended diagnostics tests including [^11^C]-methionine-positron emission computed tomography [^11^C]-methionine or [^11^C]-choline positron emission tomography/computed tomography [^11^C]-choline (PET/CT), respectively.

In some studies, the focused surgical approach has proven to result in similar success rates compared to the conventional bilateral exploration [10]. In contrast, there is also evidence that negative preoperative imaging correlates with reduced success rates of surgical exploration [11]. Moreover, according to different studies a more convincing argument for extended localization diagnostics is that a focused approach is associated with reduced morbidity, with significantly decreased rates of recurrent nerve palsy, reduced permanent hypoparathyroidism, reduced postoperative bleeding, and a shorter operation time [10, 12]. This warrants even extended preoperative imaging procedures to detect the PA preoperatively.

The imaging modalities used vary at different institutions according to local expertise and availability, but usually include high-resolution neck ultrasound, radionuclide studies, and computed tomography (CT). All these imaging modalities have been described as relatively sensitive and it has been assumed that congruent results of different imaging modalities increase the probability that the site of the adenoma can be identified quickly and smaller incision is possible during surgery [13]. Unfortunately, magnetic resonance imaging (MRI) has poor specificity and falls behind the performance of the other imaging tests.

Although there is a large body of literature on sensitivity and specificity of different imaging modalities to detect PAs prior surgery for pHPT, there are only few studies that started to create an algorithm for a sequential and rational preoperative diagnostic imaging sequence [14, 15]. Furthermore, it is not even clear whether more preoperative localization diagnostics necessarily increase the reliability to detect the PA.

Therefore, we performed a single-center retrospective analysis from surgical patients to systematically re-evaluate the diagnostic algorithms performed previously in order to improve the approach for preoperative localization diagnostics in patients with pHPT.

## Material and methods

### Setting and study design

A retrospective single-center analysis of all patients that underwent neck surgery because of primary hyperparathyroidism was carried out. Inclusion criteria were the diagnosis of primary hyperparathyroidism and clinical indication for surgery according to current guidelines [1]. Exclusion criteria were the presence of secondary or tertiary hyperparathyroidism and recurrent/persistent hyperparathyroidism. All patients with hyperparathyroidism between January 1st of 2005 and August 31st of 2021 were extracted from the local database (SAP) of our hospital after ethical approval (No. 20190205 01).

### Data acquisition

Clinical data (patient baseline characteristics: age, sex, symptoms, ASA score; parathyroid surgery with preoperative localization of the PA; serum chemistry: parathyroid hormone (PTH), calcium, phosphate, vocal cord mobility, imaging results; surgery: procedure, time, intraoperative PTH values; histopathological findings; complications: bleeding, hypocalcemia, paresthesia, recurrent laryngeal nerve palsy; postoperative parameters: serum calcium, PTH) of patients fulfilling the inclusion criteria were retrieved from the local database.

The operation (parathyroidectomy) was defined as successful, if the intraoperative PTH value had fallen to 10% of the baseline value or intraoperative PTH value < 35 pg/ml after 15 min [16, 17]. In case of inconsistencies, the original patient health record was reviewed, and missing data were included. We analyzed different factors like sex and gender as well as factors, which could have influenced the preoperative diagnosis. We defined obesity grade I: BMI 30.0–34.9, grade II: BMI 35.0–39.9, grade III: BMI 40.0–44.9.

### Preoperative localization studies

Different localization modalities were applied to patients in varying combinations including ultrasound of the neck, [^99m^Tc]-sestamibi planar scintigraphy and single-photon emission computed tomography/computed tomography (SPECT/CT), [^11^C]-methionine or [^11C^]-choline PET/CT, or magnetic resonance imaging (MRI). Ultrasound was performed by an experienced specialist in nuclear medicine, endocrinology, or endocrine surgery, respectively. The imaging procedures were performed at University Hospital of Wurzburg or by one of the collaborating specialized outpatient centers.

In the first step, all preoperatively obtained imaging procedures were reviewed according to the suspected identification of the PAs in the following categories: “left cranial,” “left caudal,” “right cranial,” “right caudal,” “others,” and “unclear.” In the second step, we evaluated the correct imaging localization, when at least the correct lateralization (right or left) of the adenoma matched to the intraoperative finding.

### Verification of the various localization modalities

Determination of the sensitivity of the different preoperative localization modalities were compared with the intraoperative “accurate” identification. This was defined by the presence of a macroscopically enlarged parathyroid gland, its histopathological confirmation in the fresh frozen section and an adequate dropping of the PTH (< 10% of the initial value or < 35 pg/dl within 15 min after extirpation of the PA [16, 17].

The preoperative identification was rated as “correct” if the predicted side (right, left, others) and altitude (cranial, caudal) matched the intraoperative finding. The sensitivity was calculated from the quotient: “all correct localization modalities” divided by “all performed localization modalities.”

### Statistics

Statistical analysis was performed with using GraphPad Prism 9.0 and Microsoft Office Excel 2010 were used for graphical presentation and additional analysis. Categorical variables were calculated with the chi^2^ test. *p* values *p* < 0.05 were considered significant.

## Results

### Patients’ characteristics

A total of 658 patients diagnosed with “primary hyperparathyroidism” were recorded in the time interval between 2005 and 2021 at the Department of Surgery, University Hospital Wurzburg. The majority of patients with pHPT (95.4%; 628/658) was operated for the first time whereas 30/658 patients (4.0%) suffered from recurrent disease (*n* = 26) or from persistent pHPT (*n* = 4). A multigland disease was diagnosed in 8.1% of the patients. Only patients with primary diagnosed pHPT were included in the following analyses.

Mean age of patients at primary diagnosis was 58.0 ± 13.2 years (range 13–93 years). The majority of patients (444/628; 70.7%) was female. Female patients were significantly older with a mean age of 59.1 ± 13 (range 13–93) at the time of diagnosis compared to male patients that were 55.9 ± 13.4 (range 18–87; *p* < 0.0056). The mean ASA for all of these patients was 2.2 ± 0.47 (range 1–3) with a mean BMI of 27.9 ± 6.1 kg/m^2^ (range 16.5–63.3) (Table 1).

**Table 1:** Patients characteristics. *p* < 0.001 male vs. female, unpaired *t*-test

		Median (range)	Mean ± SD
Basic characteristics	Sex		
	Male Female	29.3% (184/628) 70.7% (444/628)	
	Age at diagnosis (years) Male Female	58 (13–93) 57 (18–87) 59 (13–93)	58.0 ± 13.2 55.9 ± 13.4 59.1 ± 13.2
	ASA	2 (1–3)	2.2 ± 0.47
	BMI (kg/m^2^)	27 (16.5–63.3)	27.9 ± 6.1
Preoperative parameters	Calcium (mmol/l)	2.9 (2.0–3.7)	2.90 ± 0.2
	PTH (pg/ml)	130 (9.5–2373)	176.1 ± 185.2
Postoperative parameters	Calcium (mmol/l)	2.4 (1.8–3.3)	2.4 ± 0.2
	PTH (pg/ml)	12.3 (2.2–337)	24.7 ± 38.1
	Hospital stay (d)	4 (2–113)	5.3 ± 5.7

According to their diagnosis preoperative mean total calcium values amounted to 2.9 ± 0.2 mmol/l (range 2.0–3.7) and PTH was 176.1 ± 185.2 pg/ml (range 9.5–2373) in all patients. Some patients were treated preoperatively with bisphosphonates, because of high hypercalcemia or hypercalcemic crisis, so that their preoperative calcium and PTH levels were already lowered.

Postoperatively, mean total calcium values were significantly reduced to 2.4 ± 0.2 mmol/l (range 1.8–3.3; *p* < 0.0001). Similarly, PTH values were also significantly reduced to 24.7 ± 38.1 pg/ml (range 2.2–337; *p* < 0.0001) in the first postoperative measurement. Mean hospital stay was 5.3 ± 5.7 d (2–113) (Table 1). The patient, who had a hospital stay of 113 days, was a patient with a complicated course after abdominal surgery. During tracheostomy which was necessary due to long-term intensive care unit stay, thyroidectomy and parathyroidectomy were performed simultaneously because of multinodular goiter and pHPT.

In our cohort for 615 of 628 patients (97.9%), PA was successfully removed. To localize PA, neck ultrasound was carried out preoperatively in 91.7% (576/628). [^99m^Tc]-Sestamibi scintigraphy was performed in 560/628 (89.2%), whereas [^11^C]-methionine/[^11^C]-choline PET/CT was performed in 75/628 (11.9%) and MRI were performed in 19/628 (3%). Seventy-two patients had only one examination to localize PA. Four hundred seventy-two patients received two imaging procedures. Most of the patients (459/628; 73.1%) received a combination of neck ultrasound and [^99m^Tc]-sestamibi scintigraphy. Forty-six patients received three or more imaging procedures to localize PA preoperatively.

### Comparison of preoperative localization procedures with the intraoperative detection of PA

First, we compared the number of matches between preoperative localization diagnostics and intraoperative findings. As outlined above, the adequate intraoperative identification of the PA was assumed, when frozen section confirmed the presence of hyperplastic parathyroid tissue and the intraoperative PTH values had dropped significantly. At a first glance, we found that the correlation of preoperative imaging with intraoperative finding was extraordinarily low (cervical ultrasound 37%, [^99m^Tc]-sestamibi scintigraphy 39.9%, [^11^C]-methionine/[^11^C]-choline PET/CT 29.4%). We assumed that one reasonable explanation for this may be that the description of the localization by the surgeon is the localization of the PA relative to the laryngeal nerve and the inferior thyroid artery, whereas the imaging description resulted out of the relation of the PA to its surrounding macroscopic structures. Therefore, we decided to define a correct imaging procedure when at least the correct lateralization (location left/right) of the adenoma matched to the intraoperative finding. This may be useful in future interdisciplinary discussions when the results of preoperative imaging procedures are evaluated in detail.

Following this, we found that preoperative localization matched in 66.0% (380/576) independent from utilized imaging techniques. For neck ultrasound, localization matches in 70.8% (240/339), for ^99m^Tc]-sestamibi scintigraphy in 42.9% (240/560), for [^11^C]-methionine/[^11^C]-choline PET/CT in 77.3% (58/75) to the intraoperative localization of the PA. Unusual localizations of PA were found in 2.4% (15/638) of patients. These were identified in the mediastinum, intrathyroidal, or paraesophageal, and were appropriately localized by neck ultrasound 7/14 (50%), in [^99m^Tc]-sestamibi scintigraphy 4/15 (26%) or in [^11^C]-methionine/[^11^C]-choline PET/CT 4/4 (100%).

### Retrospective stratification of the diagnostic algorithm in patients according to the results of neck ultrasound

In the next step, we assigned the patient cohort group to two groups. The first group was defined as the patients with a PA identified in the preoperative neck ultrasound (Table 2). From the 441 patients in this group, the majority of 89.5% (395/441) had an additional [^99m^Tc]-sestamibi scintigraphy in the following. In 78.5% of patients, this led to the diagnosis of a suspected adenoma site which was correctly identified in 47.3% of patients (187/395) of patients. [^11^C]-Methionine/[^11^C]-choline PET/CT was carried out in 9.7% (40/441) in the subcohort of patients with a suspected adenoma in neck ultrasound. [^11^C]-Methionine/[^11^C]-choline PET/CT led to the localization of a PA in 87.5% (35/40). This site was correctly identified and confirmed during surgery in 75.0% (30/40). Finally, MRI scans were carried out in 1.8% (8/441) in this group which led to a suspected localization of the PA in 62.5% (5/8) which was confirmed during surgery in all cases (100%).

**Table 2:** Preoperative imaging is shown

		Neck ultrasound performed 91.7% (576/628)	
		PA visible 76.6% (441/576)	PA not visible 23.4% (135/576)
SESTA MIBI scan	PA suspected	78.5% (311/395)	49.2% (64/130)
	PA correctly identified	47.3% (187/395)	25.4% (33/130)
PET-CT	PA suspected	87.5% (35/40)	94.0% (32/34)
	PA correctly identified	75.0% (30/40)	79.4% (27/34)
MRI	PA suspected	62.5% (5/8)	14.3% (1/7)
	PA correctly identified	62.5% (5/8)	0% (0/7)

The second group of patients was defined as the patients in which neck ultrasound was unable to localize PA (135/567). In this group, [^99m^Tc]-sestamibi was carried out in 96% (130/135) of cases which led to a suspected localization of a PA in 49.2% (64/130). This was correctly identified intraoperatively in 25.4% of cases (33/130). In 34 of 135 (25%), patients with negative ultrasound had [^11^C]-methionine/[^11^C]-choline PET/CT in which 94% (32/34) led to the suspected localization of a PA. This was correctly identified during surgery in 27/34 patients (79.4%). MRI scans was performed in 7 patients. Out of these, in one case, PA was suspected (14.3%) but was not correctly identified intraoperatively.

**Fig. 1:**
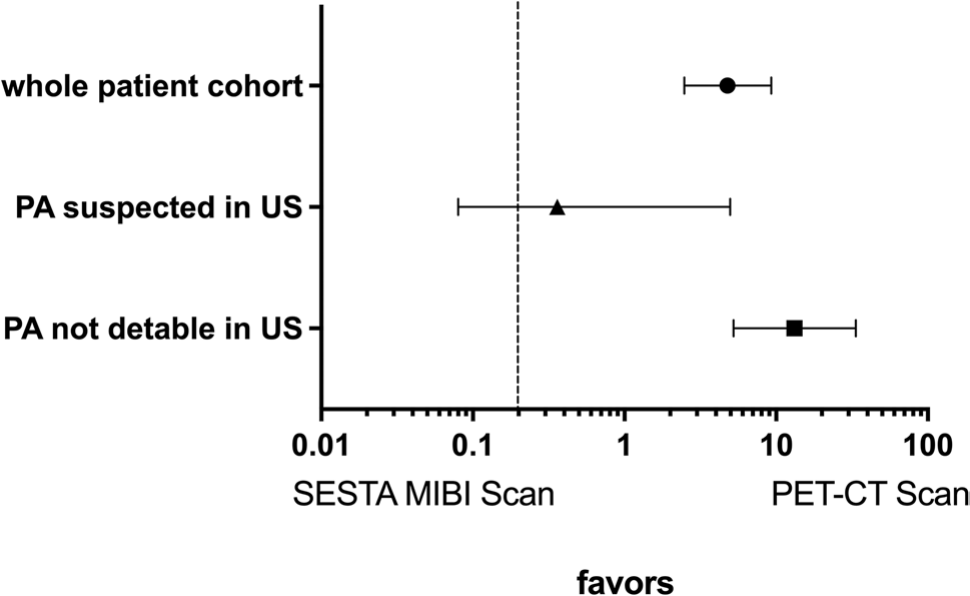
ODD ratios and 95% confidence intervals were calculated to detect the reliability of [^99m^Tc]-sestamibi compared to PET/CT

We analyzed the odds ratios (OR) to statistically compare the reliability of [^99m^Tc]-sestamibi scintigraphy compared to [^11^C]-methionine/[^11^C]-choline PET/CTs. In the whole patient cohort, the OR was 4.77 (95% CI 2.48–9.27) favoring the use of [^11^C]-methionine/[^11^C]-choline PET/CT compared to [^99m^Tc]-sestamibi scans to appropriately detect PA. This was comparable in the group of patients in which PA had been suspected by ultrasound (OR 3.98 95%CI 1.60–9.70). The diagnostic advantage was dramatically more pronounced in the group of patients in which ultrasound did not suspect PA since OR was 13.23 (95% CI 5.24–33.56) favoring the use of [^11^C]-methionine/[^11^C]-choline PET/CT compared to [^99m^Tc]-sestamibi to successfully detect PA (Fig. 1).

### Factors influencing preoperative diagnosis

Age and sex did not affect the rate of successful identification of PA in different imaging modalities. Regarding ultrasound, increased BMI negatively affected the success rates to detect PA. Patients with obesity grade I showed a matching ultrasound in 65.9% (56/85 patients), in 43.8% (18/35 patients) with obesity grade II and in 42% (21/50 patients) with obesity grade III (with correlation coefficient of *r* = 0.806). This correlation was less pronounced for [^99m^Tc]-sestamibi (*r* = 0.591) and not evident for PET-scans (*r* = 0.197).

### Surgical outcome and perioperative complications

For cases in which preoperative diagnostics to localize PA matched to the intraoperative findings, the duration of surgery was significantly shorter than with incorrect localization: 96 ± 50 min (range 14–292 min) vs. 122 ± 54 (range 19–305 min; *p* < 0.001). This includes the time until the intraoperative PTH measurements confirmed the significant drop of PTH levels, which ranges usually between 25 and 35 min at our institution. An additional factor that prolonged the duration of surgery was when thyroid surgery had been performed during the same procedure, which was the case in 42.5% (267/628) of patients. The mean time for surgery was 136 ± 58 min in those cases.

Overall, 4/628 (0.6%) patients had already a preexisting recurrent laryngeal nerve palsy (RLN) on the ipsilateral side where surgery was carried out. In the remaining fraction, for 19/624 (3.0%) of the patients a pathological intraoperative neuromonitoring was detected from which 16/624 patients (2.6%) presented with transient RLN palsy. Focused operation of the PA was associated with a decreased rate of transient RLN palsy of 1.7% (6/361; *p* = 0.129). If bilateral exploration or additional thyroid surgery was performed, the rate of transient RLN was 10/267 (3.7%). Persistent RLN was seen in 0.3% (2/624) of the patients, which both had simultaneous surgery on the thyroid gland. Interestingly, in the group of patients with transient RLN palsy, the rate the successful detection of PA (independent of the amount of imaging) was associated with a reduced rate of transient RLN palsy (successful preoperative imaging 37.5% (6/16); unsuccessful preoperative imaging: 62.5% (10/16); *p* = 0.542). None of the differences was significant which may be explained by the overall small number of complications.

Postoperative hypocalcemia was detected in 4.3% (27/628) of all patients. The majority of these patients (63%; 17/27) had bilateral exploration or simultaneous thyroid surgery, whereas all other patients (37%; 10/27) had a focused operation (*p* = 0.346). We assume that these changes were only transient although the retrospective database did not include the long-term follow-up of calcium in all of these patients. Postoperative re-bleeding needing surgical revision occurred in 9/628 (1.4%) of the patients. This appeared neither to be affected by the extent of surgery nor by anticoagulation medication.

## Discussion

The present study provides a retrospective overview on the effectiveness of imaging procedures to detect parathyroid adenoma in a single-center cohort of 628 patients with primary hyperparathyroidism. The data suggest that the value of [^99m^Tc]-sestamibi scintigraphy is low when previous ultrasound failed to identify the localization of PA. Our results in a subgroup of 34 patients indicate that then [^11^C]-methionine/[^11^C]-choline PET/CT are much more efficient and should be carried out in the next step when a focused surgical approach is intended.

With a median age of 58.0 years and a gender distribution of 70.7% female patients, as well as the preoperative values of calcium (2.9 mmol) and PTH (178 pg/ml), the patient cohort is comparable to other studies that were published previously [18–20]. Similarly, the amount of imaging to detect parathyroid adenoma before surgery in this cohort is comparable with other studies in the literature [21–23]. In our patient cohort, PA was removed successfully in 97.9% of the patient which corresponds to the high success rates of surgery for the treatment of primary hyperparathyroidism [24]. Overall, 2.6% patients had a transient RLN (recurrent laryngeal nerve) palsy. We recognized a trend of more pathological IONM (intraoperative neuromonitoring) as well as transient RLN palsy in the cases where bilateral explorations and/or additional thyroid surgery was performed. This trend was also confirmed in an augmented rate of transient hypocalcemia that amounted 4.3% in the whole cohort. In previous studies, the rate of postsurgical hypocalcemia was reported to occur in approximately 12.5% [25]. The low rates of postsurgical hypocalcemia in our cohort may be explained by the prophylactic use of activated vitamin D analogues and calcium medication during the first days after surgery at our center which is gradually terminated within 3–5 days after parathyroid surgery. Nonetheless, the trends observed for reduced laryngeal nerve palsies and reduced hypocalcemia rates argue to perform a focused approach for parathyroid surgery which has also been proposed in the literature before [25].

In summary, we assume that the current cohort presented in this retrospective analysis represents the common medical care situation in Germany and is therefore suitable for conclusions on the real-life value of preoperative imaging procedures to detect PA in primary hyperparathyroidism.

### Localization studies were compared to the intraoperative localization of parathyroid adenoma

A number of studies analyzed preoperative localization diagnostics without detailed evaluation and correlation with the intraoperative localization of the PA. Most commonly, the histopathology reports were screened to verify the successful excision of the parathyroid adenoma [26–28]. One strength of the current study is that we compared in detail the preoperative results of the imaging techniques with the intraoperative localization of the parathyroid adenoma. Therefore, we do not only report here whether different imaging procedures describe the adenoma but verify the value of the description for the surgeon.

As already outlined in the result part, we decided to define the results of an imaging procedure as a match to the intraoperative localization when at least the correct lateralization of the adenoma matched to the intraoperative finding.

### The value of [^99m^Tc]-sestamibi scintigraphy to detect parathyroid adenoma needs to be re-defined

In the majority of patients, neck ultrasound was successful to localize PA preoperatively. However, in 23.4%, ultrasound was unable to detect PA which roughly corresponds to previous data, in which ultrasound had a sensitivity of 85% [29]. Usually, in the latter cases, [^99m^Tc]-sestamibi scans are performed to exclude an aberrant location of the PA on the one hand and/or to enable a focused approach by detecting the localization of the PA on the other hand [2]. The advantage of the [^99m^Tc]-sestamibi scintigraphy is the broad availability and the standardization of this diagnostic method in nuclear medicine [9]. The main finding in our cohort was that [^99m^Tc]-sestamibi scintigraphy led to a high rate of suspects on PA when ultrasound had localized the PA before but the localization was only correctly identified intraoperatively in 47.3% of cases.

As outlined above, in cases in which PA is not visible in ultrasound, there is usually the need for an additional imaging. In this group of patients, in our cohort, we found that [^99m^Tc]-sestamibi correctly identified/localized PA only in 25.4% of patients. This clearly shows [^99m^Tc]-sestamibi is not useful to reliably detect parathyroid adenoma, especially in ultrasound negative PAs. One reason for the low rate of 25.4% correctly identified PA could be that most of the [^99m^Tc]-sestamibi scans were performed by different collaborating specialized outpatient centers. This includes patients in which the SPECT technique was applied which was reported to increase the detection rate to up 80–90% [30, 31]. Due to the large number of diagnostics carried out in outpatient centers, it was not specified in our retrospective database in all cases whether SPECT had been applied when [^99m^Tc]-sestamibi scintigraphy was carried out. Therefore, we did not include data and analysis on this here. Nonetheless, in a prospective direct comparison, the SPECT technique was clearly inferior to [^11^C]-choline PET/CT which supports our current observations [32].

### The use of [^11^C]-methionine/[^11^C]-choline PET/CT PET/CT is beneficial for patients when ultrasound fails to detect parathyroid adenoma

When we focused on the group, in which previous ultrasound had not been successful to detect PA, we found that [^11^C]-methionine/[^11^C]-choline PET/CT correctly localized PA in 79.4% of patients. The advantage of [^11^C]-methionine/[^11^C]-choline PET/CT compared to the use of MIBI-Scans is underlined by the odd’s ratio showing the strongest benefit for patients in which ultrasound had not detected PA before. Our results are supported by a previous study in which for 25 patients with negative ultrasound and [^99m^Tc]-sestamibi scan a [^18^F]-choline PET/CT detected PA with a sensitivity of 91.3% and avoided bilateral cervical exploration in 75% of the cohort [33]. In a study by Smaxwil et al., the excellent results of choline PET have been pointed out previously. For the 454 included patients, [^18F^] fluoro-ethylcholine PET/CT and 4D were carried out in ultrasound and [^99m^Tc]-sestamibi “double-negative” PAs (*n* = 109). This approach detected PA in 89.2% in the correct localization. Their overall conclusion to recommend choline PET/CT when ultrasound failed to localize PA supports our current data [34].

It has been shown before that [^11^C]-methionine/[^11^C]-choline PET/CT is superior in detecting smaller sized (> 9 mm) or multiple PAs compared to [^99m^Tc]-sestamibi scan [35]. This may explain the convincing results for [^11^C]-methionine/[^11^C]-choline PET/CT observed in our present study. In addition, the interpretation of the images was described to be easier so the rate of correctly detected PAs raised up to 96% in other patient cohorts [36–38].

Besides the obvious advantage for [^11^C]-methionine/[^11^C]-choline PET/CT to detect PA appropriately, it has been shown that especially [^11^C]-choline needs shorter acquisition times, fewer radiotracer, and there is no need to stop calcimimetic drugs. Importantly, the patients’ exposure to radiation is significantly lower when [^11^C]-methionine/[^11^C]-choline PET/CT is performed compared to MIBI-scans [39, 40]. For 11C-choline, the effective dose for an adult is 2.9 mSv, which is lower relative to [^99^mTc]-sestamibi (6.3 mSv) [2].

Finally, our study has several limitations including the retrospective character of the study and the long period of patient acquisition. Furthermore, preoperative imaging was not standardized and performed by many different clinicians.

The strength of our study is that we present a large representative cohort of patients which reflect the common medical care situation in Germany and is therefore suitable for conclusions on the real-life value of preoperative imaging procedures in the treatment of primary hyperparathyroidism.

A practical problem is that [^11^C]-methionine/[^11^C]-choline PET/CT is not universally available and restricted to a small number of centers. Currently, these will not be able to assess all of the patients with pHPT with negative ultrasound to localize PA. Another problem in Germany where this study was carried out is that health insurances pay for [^11^C]-methionine/[^11^C]-choline PET/CT only after case-by-case assessment, which is an additional limitation for the broad use of PET/CT.

## Conclusion

**Fig. 2:**
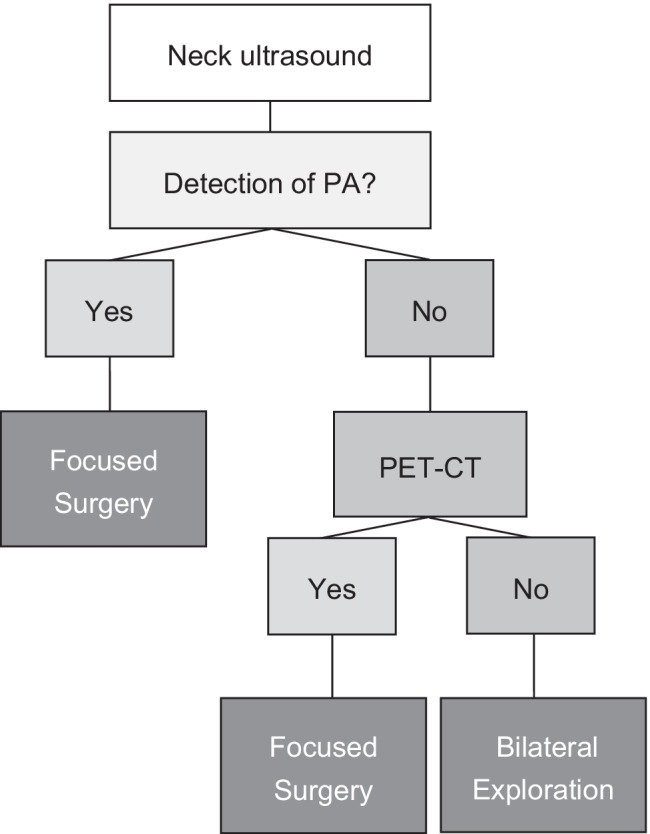
Proposed algorithm to localize PA for focused surgery. Based on our data, we suggest the use of this algorithm for the future. Limitations concerning the availability and economic considerations are outlined in the discussion. In case of detection of PA by ultrasound surgery can be performed without additional localization techniques. In cases PA is not detected by ultrasound [^11^C]-choline or [^11^C]-methionine PET/CT should be carried out instead of [^99m^Tc] sestamibi scintigraphy

In view of the results provided by the use of the [^11^C]-methionine/[^11^C]-choline PET/CT to detect PA in primary hyperparathyroidism and the potential to reduce radiation exposure to patients, we suggest the use of PET/CT instead of MIBI-scans in patients with negative ultrasound (Fig. 2). However, the limited availability of PET/CTs and economic limitations will currently make a broad practical implication of this suggestion difficult.
